# PBHMDA: Path-Based Human Microbe-Disease Association Prediction

**DOI:** 10.3389/fmicb.2017.00233

**Published:** 2017-02-22

**Authors:** Zhi-An Huang, Xing Chen, Zexuan Zhu, Hongsheng Liu, Gui-Ying Yan, Zhu-Hong You, Zhenkun Wen

**Affiliations:** ^1^College of Computer Science and Software Engineering, Shenzhen UniversityShenzhen, China; ^2^School of Information and Control Engineering, China University of Mining and TechnologyXuzhou, China; ^3^School of Life Science, Liaoning UniversityShenyang, China; ^4^Research Center for Computer Simulating and Information Processing of Bio-Macromolecules of Liaoning ProvinceShenyang, China; ^5^Academy of Mathematics and Systems Science, Chinese Academy of SciencesBeijing, China; ^6^Xinjiang Technical Institute of Physics and Chemistry, Chinese Academy of Scienceürümqi, China

**Keywords:** microbes, diseases, path-based measure, computational prediction model, association network

## Abstract

With the advance of sequencing technology and microbiology, the microorganisms have been found to be closely related to various important human diseases. The increasing identification of human microbe-disease associations offers important insights into the underlying disease mechanism understanding from the perspective of human microbes, which are greatly helpful for investigating pathogenesis, promoting early diagnosis and improving precision medicine. However, the current knowledge in this domain is still limited and far from complete. Here, we present the computational model of Path-Based Human Microbe-Disease Association prediction (PBHMDA) based on the integration of known microbe-disease associations and the Gaussian interaction profile kernel similarity for microbes and diseases. A special depth-first search algorithm was implemented to traverse all possible paths between microbes and diseases for inferring the most possible disease-related microbes. As a result, PBHMDA obtained a reliable prediction performance with AUCs (The area under ROC curve) of 0.9169 and 0.8767 in the frameworks of both global and local leave-one-out cross validations, respectively. Based on 5-fold cross validation, average AUCs of 0.9082 ± 0.0061 further demonstrated the efficiency of the proposed model. For the case studies of liver cirrhosis, type 1 diabetes, and asthma, 9, 7, and 9 out of predicted microbes in the top 10 have been confirmed by previously published experimental literatures, respectively. We have publicly released the prioritized microbe-disease associations, which may help to select the most potential pairs for further guiding the experimental confirmation. In conclusion, PBHMDA may have potential to boost the discovery of novel microbe-disease associations and aid future research efforts toward microbe involvement in human disease mechanism. The code and data of PBHMDA is freely available at http://www.escience.cn/system/file?fileId=85214.

## Introduction

It is well known that microorganisms are ubiquitously existing in the environment and occupying almost all habitats including animals and humans. The microorganisms in human body mainly refer to bacteria, fungi, viruses, archea, and protozoa (Consortium, 2012a; Sommer and Bäckhed, 2013). Accumulating evidences suggest that these microbes are mostly harmless to human, and very essential for human physiology (e.g., enhancing metabolic capability, strengthening immune system and offering protection from pathogens; Ventura et al., 2009). For example, in the adult gut, the large majority of intestinal microorganisms (10^13^–10^14^) inhabiting the gastrointestinal tract not only synthesize essential amino acids and vitamins but also improve digestion of the indigestible components to human diet (e.g., plant polysaccharides). That is why microbes could be regarded as a microbial organ coexisting within a host organ (Bäckhed et al., 2005). Therefore, constant change in the communities of microorganisms might influence human health and disease. After the first finding that microorganisms caused disease in humans in the 1800s, researchers were inspired to study the complex morphology and characteristic of human-associated microorganisms. These researches led to saving millions of lives through improved hygiene, antibiotics, and vaccinations (Dethlefsen et al., 2007). Researchers have obtained preliminary understanding of structure, composition, and function from microbiological perspective for revealing the host-microbe interactions by investigating the human indigenous microbes.

Understanding the host-microbe interactions is greatly significant in human microbiome studies. The mutualistic symbiotic relationship is naturally selected and developed over millennia by the co-evolution between humans and their symbiotic microbes. The microbial communities are greatly affected by both the genetics (Khachatryan et al., 2008; Turnbaugh et al., 2009; Goodrich et al., 2014) and the dynamic habitat environments [e.g., antibiotics (Donia et al., 2014), season (Davenport et al., 2014), diets (Muegge et al., 2011; Walker et al., 2011; Wu et al., 2011; David et al., 2014), and smoking (Mason et al., 2015)] of the host. For example, some studies suggest that high-fat maternal or postnatal diet has a potential role in shaping the offspring commensal microbial communities in human (Ma et al., 2014). Moreover, the human complex immune mechanisms have homeostatic roles to monitor and control a dynamic balance of indigenous microbial communities. Therefore, the imbalance or dysbiosis of microbial communities may directly affect the well-being of humans and even cause diseases (Neish, 2009). Advances in sequencing technology and microbiology have allowed researchers to investigate the microbiome from cohort by incorporating several complementary analyses including: alignment of the assembled sequences to the reference microbial genomes, 16S ribosomal RNA (16S rRNA) gene sequence and taxonomic profiles, and metagenomic sequencing of whole microbial community DNA or whole-genome shotgun sequencing (Consortium, 2010, 2012b). They have facilitated the identification of the relationships between the human microbiota and various diseases such as cancer (Schwabe and Jobin, 2013), autoinflammatory disease (Lukens et al., 2014), metabolic syndrome (Wen et al., 2008; Ley, 2010; Qin et al., 2012), cardiovascular disease (Koeth et al., 2013; Tang et al., 2013), and central nervous system disorder (Wang and Kasper, 2014). For examples, Brown et al. (2011) discovered that, the autoimmune subjects could be participated in triggering a type 1 diabetes associated autoimmune response. Significant differences in metabolic potential showed that these autoimmune subjects tend to have a functionally aberrant microbiome. Data analysis showed that a consortium of bacteria (i.e., *Acetonema*) which produces lactate and butyrate in a healthy gut induces a sufficient level of synthesis to maintain integrity of gut. Previous evidences (Turnbaugh et al., 2006; Musso et al., 2010) indicated that the microbial communities inhabiting in the human intestine are closely related to the pathogenesis of obesity. Zhang et al. (2009) examined the microbial 16S rRNA genes by using real-time PCR for comparing the microbial community structures of nine individuals, which were categorized into normal weight, morbidly obese and post-gastric-bypass surgery. They detected that higher numbers of both H(2)-producing *Prevotellaceae* and H(2)-utilizing methanogenic archaea are significantly enriched in the obese individuals than in post-gastric-bypass individuals or normal-weight individuals. This observation strongly supports the hypothesis that the transformation of interspecies H(2) between bacterial and archaeal species forms an important mechanism providing increasing energy uptake by the large intestine in obese individuals. Furthermore, it is well known that the human mouth harbors thousands of bacterial types constructing the complex ecosystem. The proliferation of pathogenic oral bacteria could lead to an inflammatory disease (i.e., periodontitis), which is bound to a high risk of the cardiovascular disease.

Based on the hypothesis proposed by Ma et al. (2017) that those microbes involved in phenotypically similar disease tend to be functionally similar and vice versa, developing a computational model could facilitate the identification of novel disease-related microbes by inferring the most potential microbe-disease associations on a large scale (Chen et al., 2015a,b; Chen, 2016; Chen et al., 2016a,b,c). The increasing studies provide valuable insights into the underlying relationships between dysfunction of human microbes and human diseases, which are helpful for exploring the pathogenesis of human diseases. However, traditional cultivation-based approaches which researchers previously heavily relied on are not only time-consuming but also laborious. Few deep surveys and available data result in a narrow knowledge of this domain, which severely restricts the development of microbiology for pathology. Considering the current understanding of microbiology, it is insufficient to advance the applications in diagnosis, treatment and prevention of human complex diseases. Ma et al. (2017) has currently implemented a large-scale text mining to establish the Human Microbe-Disease Association Database (HMDAD) by collecting the microbe-disease associations from previous literatures. HMDAD database currently integrates 483 disease-microbe entries, which could be an information resource for the Microbe-Disease Association Prediction.

In this paper, we developed the novel computational model of Path-Based Human Microbe-Disease Association prediction (PBHMDA) based on a heterogeneous network composed of the known microbe-disease associations and Gaussian interaction profile kernel similarity for microbes and diseases. A special depth-first search algorithm was further adopted to calculate the total scores for prioritizing the most likely disease-related microbes. Three evaluation frameworks, including global leave-one-out cross validation (global LOOCV), local leave-one-out cross validation (local LOOCV) and 5-fold cross validation (5-fold CV), were implemented to evaluate the prediction performance of PBHMDA. Despite of depending on the sole information source (i.e., the known microbe-disease associations in HMDAD database), PBHMDA achieved the reliable performance in the frameworks of both global and local LOOCV (AUCs of 0.9169 and 0.8767, respectively) and 5-fold CV (average AUC value of 0.9082 ± 0.0061). We also implemented case studies of liver cirrhosis, type 1 diabetes and asthma for the further evaluation. 9, 7, and 9 out of disease-related microbes predicted in the top 10 obtained experimental confirmations based on previous literature evidences, respectively. These prediction results totally demonstrated the reliable prediction accuracy of PBHMDA, which could be considered as a promising data-mining tool to boost the identification of underlying microbe-disease associations. In human health, this prediction tool will provide insights from microbiology and disease understanding to identify biologically relevant biomarkers and ultimately inform targeted therapeutic intervention.

## Materials and methods

### Human microbe-disease associations

Human Microbe-Disease Association Database (HMDAD) database (http://www.cuilab.cn/hmdad) has collected 450 verified human microbe-disease associations including 292 microbes and 39 diseases (Ma et al., 2017). The names of Microorganism were mostly curated at the genus level because lots of microbiome studies using 16s RNA sequencing only provided the genus-level information. We defined the adjacency matrix of microbe-disease association network as variable *Y*, i.e., if microbe *m(i)* was identified to be associated with disease *d(j)*, the entity *Y(i,j)* was equal to 1, otherwise 0. Two variables *nm* and *nd* denoted the numbers of microbe and disease investigated in this study, respectively.

### Gaussian interaction profile kernel similarity for diseases

Under the assumption that similar diseases tend to be associated with the functionally similar microbes and therefore share the similar interaction and non-interaction patterns with microbes, we utilized the Gaussian kernel for the interaction profiles of diseases to calculate disease similarity from the known microbe-disease associations. This process could be described into the following two steps. First, the interaction profile of disease *d(i)* is denoted by a binary vector *IP(d(i))* for representing whether disease *d(i)* is associated with each microbe or not, i.e., the *i*^*th*^ column of the binary adjacency matrix *Y*. Second, the kernel for two diseases *d(i)* and *d(j)* is defined to calculate Gaussian kernel similarity based on their interaction profiles, defined as follows (Chen and Yan, 2013):

(1) $$\begin{array}{r} KD\left(d\left(i\right),d\left(j\right)\right)\;=\;\exp\left(-\gamma_{d}{\left||IP\left(d\left(i\right)\right)\;-\;IP\left(d\left(j\right)\right)|\right|}^{2}\right) \end{array}$$

(2) $$\begin{array}{r} \gamma_{d}\;=\;{\gamma \prime }_{d}/\left(\frac{1}{nd}\overset{nd}{\underset{i=1}{\sum }}{\left||IP\left(d\left(i\right)\right)|\right|}^{2}\right) \end{array}$$

where the parameter γ_*d*_ controls the kernel bandwidth, which could be obtained through normalizing a new bandwidth parameter γ_*d*_ by the average number of associations with microbes per disease. Although this new bandwidth parameter γ_*d*_ could be better replaced by other value according to the further cross validation, here we set γ_*d*_ = 1 for simplicity according to previous relevant research (Chen and Yan, 2013). *KD* is a symmetric matrix whose entity *KD(i,j)* denotes the Gaussian interaction profile kernel similarity between disease *d(i)* and disease *d(j)*. In the framework of cross validation, Gaussian interaction profile kernel similarity for diseases and microbes needed to be recalculated when we removed the left-out known microbe-disease associations.

### Gaussian interaction profile kernel similarity for microbes

Similarly, based on the assumption that the microbes which share the similar diseases tend to be functionally similar, the Gaussian interaction profile kernel similarity for microbe also could be calculated in the similar way as diseases (Chen and Yan, 2013):

(3) $$\begin{array}{r} KM\left(m\left(i\right),m\left(j\right)\right)=\exp\left(-\gamma_{m}{\left||IP\left(m\left(i\right)\right)\;-\;IP\left(m\left(j\right)\right)|\right|}^{2}\right) \end{array}$$

(4) $$\begin{array}{r} \gamma_{m}\;=\;{\gamma \prime }_{m}/\left(\frac{1}{nm}\overset{nm}{\underset{i=1}{\sum }}{\left||IP\left(m\left(i\right)\right)|\right|}^{2}\right) \end{array}$$

where *KM* is the Gaussian interaction profile kernel similarity for all investigated microbes and the parameter γ_*m*_ regulates the kernel bandwidth, which should be obtained through normalizing a new bandwidth parameter ${\gamma \prime }_{m}$ (${\gamma \prime }_{m}$ = 1) by the average number of associations with diseases per microbe.

### Heterogeneous interlinked network construction

Based on the known microbe-disease associations and Gaussian interaction profile kernel similarity for both microbes and diseases, we constructed a heterogeneous interlinked network with these three relations, i.e., microbe to disease, microbe to microbe, and disease to disease relations. Two node sets of microbes and diseases are defined as M = {m(1), m(2), …, m(nm)} and D = {d(1), d(2), …, d(nd)}, respectively. The weight of an edge between a microbe and a disease represents the interaction or non-interaction pattern between them, i.e., if a microbe has known association with a disease, the weight of edge between them is equal to 1, otherwise 0. The weight of an edge between two microbes or two diseases represents microbe similarity or disease similarity inferred from the Gaussian interaction profile kernel similarity. To select the valuable associations and significantly reduce runtime, we set a threshold *T* to eliminate weak correlations in the network. Namely, those edges with assigned weights smaller than *T* are not shown in the network. In this way, a heterogeneous interlinked network can be constructed.

### PBHMDA

**Figure 1:**
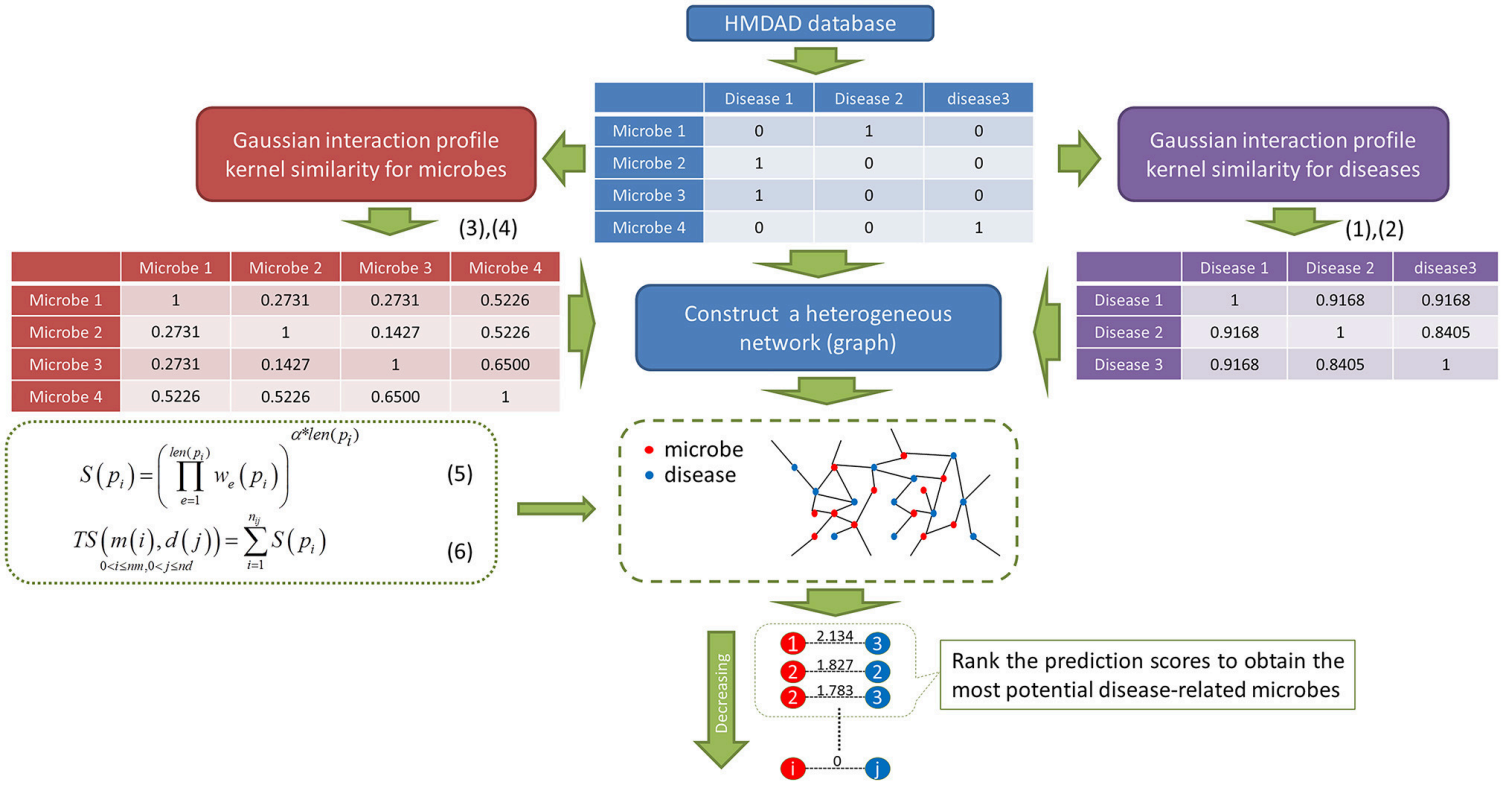
**Flowchart of PBHMDA**. Based on the heterogeneous network constructed by the integration of three different networks, we implemented a special depth-first search algorithm to identify the potential microbe-disease associations. (*nm* and *nd*: the numbers of microbes and diseases, α: the decay coefficient)

As showed in Figure 1, PBHMDA is a novel path-based prediction model for inferring potential microbe-disease associations. In our method, all paths between a microbe and a disease are traversed to calculate their association scores. These paths are not allowed to have a cycle, which means there are no repeated nodes along each path. Based on the assumption that a microbe and a disease are more possibly associated when more paths are found to connect them, we therefore devised a special depth-first search algorithm to calculate the association scores for each microbe-disease pair. This algorithm keeps track of the visited nodes and ensures that no repeated nodes are visited along a specific path. So it is easily implemented as a recursive function which marks the visited nodes and then deletes the mark before returning from the recursive call. For saving time, this algorithm should be predefined a limited maximum length *L*, i.e., the edge number of each path does not exceed *L*. In general, a shorter path between a microbe and a disease should indicate a more confident association. Therefore, for a single path p_i_ between a microbe *m(i)* and a disease *d(j)*, we devised equation (5) with an exponential decay function to obtain its prediction score *S*(*p*_*i*_):

(5) $$\begin{array}{l} S\left(p_{i}\right)={\left(\prod_{e=1}^{len\left(p_{i}\right)}w_{e}\left(p_{i}\right)\right)}^{\alpha *len\left(p_{i}\right)} \end{array}$$

where *w*_*e*_(*p*_*i*_) is the weight of the eth edge along *p*_*i*_, len(*p*_*i*_) is the length of path p_i_, and the parameter α is a decay coefficient.

In this way, all paths between a microbe *m(i)* and a disease *d(j)* are correspondingly assigned with prediction scores, which represent their association probabilities, i.e., the higher scores they get, the more likely associated they would be. By aggregating these prediction scores, the total association score between *m(i)* and *d(j)* can be obtained as follows:

(6) $$\begin{array}{l} \underset{0<i\leq nm,0<j\leq nd}{TS\left(m\left(i\right),d\left(j\right)\right)}=\sum_{i=1}^{n_{ij}}S\left(p_{i}\right) \end{array}$$

where n_ij_ indicates the number of paths between microbe *m(i)* and disease *d(j)*. Finally, the potential microbe-disease associations are ranked for prioritization. Those microbe-disease pairs in the top rank could be regarded as the most potential microbe-disease associations for further biological experimental confirmation.

## Results

### Model design

Based on the assumption that the abnormal microbes of the similar function tend to be implicated in similar diseases and vice versa, we are inspired to devise a novel computational model of PBHMDA (See Figure 1). For fully exploring potential microbe-disease associations, microbe-microbe similarity and disease-disease similarity are respectively inferred based on the Gaussian interaction profile kernel similarity. In this way, a heterogeneous interlinked network can be integrated by these three sub-networks (i.e., known microbe-disease interaction network, microbe similarity network, and disease similarity network). A special depth-first search algorithm was developed to calculate the prediction scores for each candidate disease-microbe pair based on all possible paths connecting the microbe nodes and the disease nodes. Finally, the most potential microbe-disease associations could be prioritized by ranking their aggregated scores.

### Performance evaluation

**Figure 2:**
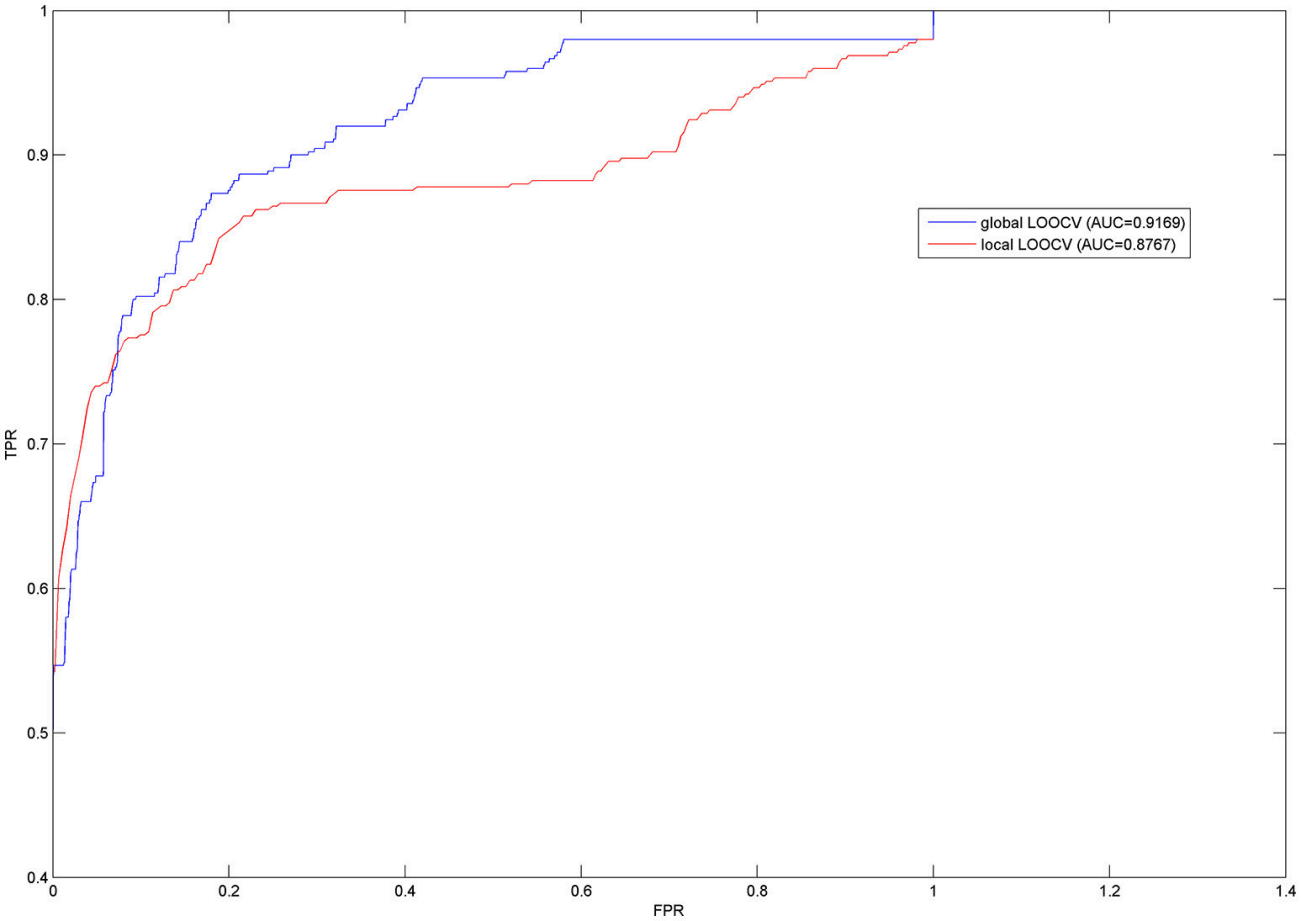
**Prediction performances of PBHMDA in the frameworks of both global and local LOOCV**.

In order to effectively evaluate the prediction performance of PBHMDA, both LOOCV and 5-fold CV were implemented on PBHMDA based on the known disease-microbe associations from HMDAD database, which collected and provided the experimentally verified human microbe-disease association dataset. In this paper, LOOCV can be divided into the following two ways: (1) For the given *j*^*th*^ disease *d(j)*, each known microbe associated with *d(j)* was left out in turns as a test sample and other microbe-disease associations were used for training model. Based on their prediction score reflecting the association probability between microbe *m(i)* and disease *d(j)*, the test sample was merely ranked in the scope of disease *d(j)* with all microbes, which had not been confirmed to have associations with disease *d(j)* in the known dataset from HMDAD database. This model was considered to make a correct prediction for microbe-disease association when the rank of the test sample exceeds the given threshold. We called this validation method of LOOCV as local LOOCV. (2) Instead of being limited in one disease, we take all investigated diseases into account simultaneously. Each known microbe-disease association was left out in turns as a test association and others for training as well. The single test association was ranked in all unverified microbe-disease associations in HMDAD database based on their prediction scores. Likewise, the test association with a higher rank than the given threshold would be considered as a successful prediction. We called this way of LOOCV as global LOOCV. Therefore, the difference between global and local LOOCV is whether all investigated diseases are considered simultaneously. Receiver-operating characteristics (ROC) curve is widely used in binary classification problems. By varying the thresholds, the true positive rate (TPR, sensitivity) versus false positive rate (FPR, 1-specificity) can be calculated to plot the ROC curves. In our approach, sensitivity refers to the percentage of the positive test samples with higher ranks than the specific threshold; specificity refers to the percentage of negative test samples with lower ranks than the specific threshold. AUC was further calculated for a numerical evaluation of prediction performance, e.g., AUC = 0.5 means a purely random result and AUC = 1 means a perfect performance. As a result, our model obtained the high AUCs of 0.9169 and 0.8767 in the frameworks of both global and local LOOCV respectively (see Figure 2).

Furthermore, 5-fold CV randomly divided all known microbe-disease associations into five disjoint groups (one group for testing in turns and other four groups for training model). For reducing bias brought by sample divisions, we implemented random divisions 100 times to evaluate the robustness of PBHMDA. We obtained the ROC curves and AUCs in the similar way as LOOCV mentioned above. As a result, the average AUC value was 0.9082 with the standard deviation of 0.0061. These prediction results fully demonstrate the strong prediction performance of PBHMDA which can provide reliable microbe candidates for future studies in the pathogenesis of human diseases.

### Prioritize novel microbe-disease associations

After demonstrating the accurate predictive capacity in the cross validation, we further utilized PBHMDA to prioritize the most potential microbes associated with all investigated diseases. These predicted results are publicly released (see Supplementary Table 1). Because of the limited current knowledge, almost all these disease-microbe associations are totally new prediction without any experimental confirmation. Therefore, it is anticipated that these prediction results could offer insights to facilitate the identification of underlying microbe-disease associations and explore the pathology from microbiological perspective.

### Case study

For further evaluating the newly proposed model, we implemented the case studies of liver cirrhosis, type 1 diabetes, and asthma on PBHMDA by observing how many predicted microbes in the top 10 were verified by experimental literatures.

Identification of more potential related microbes is helpful to understand the pathology of liver cirrhosis. In the prediction list, 9 of predicted microbes in the top 10 have been verified from experimental evidence (See Table 1). For example, researchers revealed that *Firmicutes* (1st in the prediction list) is highly enriched in the cirrhosis people (Chen et al., 2011). *Bacteroidesvulgatus* (2nd in the prediction list) was discovered to have overgrowth in non-alcoholic steatohepatitis (NASH) cirrhosis samples (Kakiyama et al., 2013). The population of *Porphyromonadaceae* (3rd in the prediction list) and *Eubacteriaceae* (4th in the prediction list) declines in a patient with worsening liver cirrhosis (Bajaj et al., 2014; van Best et al., 2015). Furthermore, an increase of *Actinobacteria* (5th in the prediction list) was found to be associated with live cirrhosis (Fouts et al., 2012). In addition, *Yersinia* (6th in the prediction list) is considered as an aggressive bacteria to selectively colonize the cirrhosis samples (Vadillo et al., 1994). *Vibrio vulnificus* (7th in the prediction list) infection has been recognized as a major cause of fatal septicemia in chronically ill patients, especially those with liver cirrhosis (Vollberg and Herrera, 1997). By sequencing of 16S rRNA genes, an abundance of *Verrucomicrobia* (8th and 9th in the prediction list) bacteria was found in the mouse with alcoholic liver disease (Yan et al., 2011; Seki and Schnabl, 2012).

**Table 1:** **For further evaluating the prediction performance of newly developed computational model, PBHMDA was applied to liver cirrhosis**.

**Rank**	**Microbe**	**Evidence**
1	Firmicutes	PMID: 21574172
2	Bacteroides vulgatus	PMID: 23333527
3	Porphyromonadaceae	PMID: 24374295
4	Eubacteriaceae	PMID: 26067771
5	Actinobacteria	PMID: 22326468
6	Yersinia	PMID: 8086556
7	Vibrio	PMID: 9120332,15842598
8	Verrucomicrobia bacterium MS-F-88	PMID: 21254165
9	Verrucomicrobia	PMID: 22124143
10	Unidentified bacterium ZF3	Unconfirmed

As a result, 9 out of predicted microbes in the top 10 have been verified by previous experimental literatures. (PMID: PubMed Unique Identifier)

In the prediction list of type 1 diabetes, 7 of predicted microbes in the top 10 have been validated by experimental literatures (see Table 2). Among them, a significant increase of *Enterobacteriaceae, Ruminococcus, Coprococcus, Clostridium* and *Faecalibacterium prausnitzii* (1st, 3rd, 7th, 8th, and 9th in the prediction list, respectively) may result in a disturbance in the ecological balance, which could cause type 1 diabetes (Murri et al., 2013; Soyucen et al., 2014; Tejesvi et al., 2016). The level of *Pasteurellaceae* (4th in the prediction list) in disease group is remarkably lower than those in healthy group (Qi et al., 2016). *Megasphaera* (10th in the prediction list) was found to only occur in patients with type 1 diabetes (Tejesvi et al., 2016).

**Table 2:** **PBHMDA was applied to type 1 diabetes**.

**Rank**	**Microbe**	**Evidence**
1	Enterobacteriaceae	PMID: 24475780
2	Streptococcaceae	Unconfirmed
3	Ruminococcus	PMID: 23433344
4	Pasteurellaceae	PMID: 27231166
5	Haemophilus parainfluenzae	Unconfirmed
6	Dorea	Unconfirmed
7	Coprococcus	PMID: 26718942
8	Clostridium	PMID: 23433344
9	Faecalibacterium prausnitzii	PMID: 20613793
10	Megasphaera	PMID: 26718942

*As a result, 7 out of predicted microbes in the top 10 have been verified by previous experimental literatures*.

In the prediction list of asthma, 9 of microbes predicted in the top 10 have been confirmed by experimental literatures (see Table 3). For example, *Firmicutes, Lachnospiraceae, Veillonella* and *Actinobacteria* (1st, 3rd, 4th, and 9th in the prediction list) present lower proportions in asthmatic patients (Marri et al., 2013; Lee et al., 2014; Park et al., 2014). The decrease of *Lactobacillus* (2nd in the prediction list) in the asthmatic samples is anticipated to be used for the prevention of asthma (Yu et al., 2010). *Bacteroides* (5th in the prediction list) colonization could be considered as an early indicator to judge whether children at the age 3 weeks potentially have asthma later in life (Vael et al., 2008). *Bacteroidaceae* and *Fusobacterium* (6th and 8th in the prediction list) is much more abundant in asthmatic patients relative to healthy people (Dang et al., 2013; Qiu et al., 2016). Based on the effect of *Streptococcus pneumonia* (7th in the prediction list) on the development of asthma, effective immunomodulatory therapies are hoped to be presented (Preston et al., 2007).

**Table 3:** **PBHMDA was applied to asthma**.

**Rank**	**Microbe**	**Evidence**
1	Firmicutes	PMID: 23265859
2	Lactobacillus	PMID: 20592920
3	Lachnospiraceae	Lee et al., 2014
4	Veillonella	PMID: 25329665
5	Bacteroides	PMID: 18822123
6	Bacteroidaceae	Qiu et al., 2016
7	Streptococcus	PMID: 17950502
8	Fusobacterium	Dang et al., 2013
9	Actinobacteria	PMID: 23265859
10	Eubacterium	Unconfirmed

*As a result, 9 out of predicted microbes in the top 10 have been verified by previous experimental literatures*.

## Discussion

In the past few years, with the advance of sequencing technology and microbiology, the microbes inhabiting in human body have been confirmed to play a significantly important role in the development and progression of human diseases. Increasing disease-related microbes which have been identified help researchers further understand the mechanisms of the pathogenesis from the perspective of microbe. We here presented a novel computational model of Path-Based Human Microbe-Disease Association prediction (PBHMDA) based on the known associations derived from the HMDAD database. The known microbe-disease associations and Gaussian interaction profile kernel similarity for both microbes and diseases are combined to construct a heterogeneous interlinked network, which can be utilized to obtain the prediction scores for each candidate microbe-disease pair by a special depth-first search algorithm. PBHMDA achieved the reliable AUCs of 0.9169 and 0.8767 in the evaluation frameworks of global and local LOOCV, respectively. Moreover, the proposed model was also evaluated with 5-fold CV for 100 times and the average AUC value of 0.9082 ± 0.0061 was obtained. The evaluation performance fully demonstrated that PBHMDA had satisfactory prediction capability in spite of using the single information source (i.e., known microbe-disease associations). Furthermore, as a global measure model, PBHMDA can simultaneously predict novel microbes associated with all investigated diseases.

Many potential microbes in the top rank were predicted to have associations with digestive system and respiratory diseases, so we implemented the case studies of liver cirrhosis, type 1 diabetes, and asthma for further evaluation of the proposed model. These three important diseases have been reported to not only have a strong link with microbes, but also severely threaten human health for these years. In 2010, liver cirrhosis was ranked as the fifth leading cause of death for people aged between 45 and 64 in the United States, killing about 20,000 people (Miniño, 2011). Previous research (Liu et al., 2012) showed that human liver is closely related to the gut in biological function and pathogenic process. The disruption of the ecological balance of the gut microbiota may lead to severe liver damage, including cirrhosis. For example, the inadequacy of *Bifidobacterium* in gut could trigger cirrhosis. A significant difference of both population and bacterial community structure of *Enterobacteriaceae* was detected between cirrhosis group and healthy group. Identification of more potential related microbes is helpful to understand the pathology of liver cirrhosis. Moreover, Type 1 diabetes is a form of diabetes mellitus typically beginning in child and young adults. It results from the lack of insulin. The prevalence of type 1 diabetes has risen dramatically over the last 50 years. In 2009, 166,984 youths have been diagnosed with type 1 diabetes in America (Chiang et al., 2014). However, the cause of type 1 diabetes still remains unknown. The prevention methods of type 1 diabetes have not been found so far. Recently, several studies (Wen et al., 2008; Giongo et al., 2011) have shown that a significant change in human microbial environment is involved in the development of autoimmune disorders, which often lead to type 1 diabetes. The hundred trillion bacteria residing in human gut establish a symbiotic relation with the host and influence many aspects of metabolism, physiology, and immunity. Through the metagenome analysis, *Actinobacillus, Erwinia* and *Coprobacillus* in disease samples are much more abundant than healthy samples', which reveals that these three types of microbes may be involved in triggering type 1 diabetes (Brown et al., 2011). Furthermore, Asthma is a common long term inflammatory disease of the airways of the lungs characterized by recurrent attacks of wheezing and breathlessness. Asthma often begins in childhood. Disease prevalence is higher amongst women, families below the poverty line, and people of multiple races compared to other groups (Akinbami et al., 2009). Asthma is difficult to be diagnosed because of inherent heterogeneity across asthmatic patient populations and the multiple contributory factors such as environmental exposures and lung function. Up to now, researchers have not found a treatment to cure asthma. In 2013, it caused about 489,000 deaths and 242 million diagnosis cases all over the world, which significantly increases since 1960s (Anandan et al., 2010; GBD 2013 Mortality and Causes of Death Collaborators, 2015). Although the causes of asthma are clearly multifactorial, the increasing evidences support that host-microbe interactions may be involved in the pathogenesis of asthma. Multiple studies of asthmatic patient cohorts using distinct microbiome platforms have reported the presence of a diverse microbial community in the airways of these patients (Goleva et al., 2013). Thus, the imbalance or dysbiosis of these microbial communities may directly cause human diseases or disorders. Based on the previous literatures, 9, 7, and 9 of predicted microbes in the top 10 were experimentally verified to be associated with liver cirrhosis, type 1 diabetes and asthma in the case studies, respectively. The prediction accuracy of PBHMDA could be demonstrated through these three case studies. Meanwhile, this prediction is well adapted to microbial community diversity and reflects the characteristics of different kinds of diseases. In conclusion, these results demonstrate the promising performance of PBHMDA, which could be used to predict more potential disease-related microbes in the future.

Some critical factors for the reliable performance of PBHMDA could be summarized as follows. First, the HMDAD database provides known microbe-disease associations, which are reliable as a basic information resource. Second, we utilized the Gaussian interaction profile kernel similarity to accurately measure microbe similarity and disease similarity, which contribute to constructing the heterogeneous interlinked network for further introducing the novel algorithm. Last but not least, path-based algorithm can fully explore the implied topologic information in the network for predicting the underlying microbe-disease associations.

Certainly, this model is still restricted by some limitations. The prediction performance is limited by the sparse microbe-disease association network obtained from the HMDAD database. As the increasing microbe-disease associations are identified and collected, this problem could be solved in the future. Furthermore, it is unavoidable to cause bias to heavily investigated diseases and microbes considering the fact that microbe-microbe similarity and disease-disease similarity are inferred from known microbe-disease associations based on Gaussian kernel for interaction profiles of microbes and diseases. Adopting other beneficial biological datasets (e.g., microbe functional similarity and disease semantic similarity), could help us improve the quality of both microbe and disease similarity network and the prediction performance of computational models (Chen and Yan, 2014; Chen et al., 2016d). Finally, PBHMDA cannot work well for the new microbes without known associated diseases and new diseases without known associated microbes.

## Author contributions

ZH implemented the experiments, analyzed the result, and wrote the paper. XC conceived the project, developed the prediction method, designed the experiments, analyzed the result, and wrote the paper. ZZ conceived the project, implemented the experiments, and analyzed the result. HL revised the paper. GY analyzed the result and revised the paper. ZY and ZW analyzed the result. All authors read and approved the final manuscript.

## Funding

XC was supported by National Natural Science Foundation of China under Grant No. 11301517 and 11631014. ZZ was supported by the National Natural Science Foundation of China under Grant No. 61471246, Guangdong Foundation of Outstanding Young Teachers in Higher Education Institutions under Grant No. Yq2013141, and Guangdong Special Support Program of Top-notch Young Professionals under Grant No. 2014TQ01X273. HL was supported by National Natural Science Foundation of China under Grant No. 31570160 and Innovation Team Project from the Education Department of Liaoning Province under Grant No. LT2015011. GY was supported by National Natural Science Foundation of China under Grant No. 11371355 and 11631014. ZY was supported by National Natural Science Foundation of China under Grant No. 61572506 and Pioneer Hundred Talents Program of Chinese Academy of Sciences. ZW was supported by National Nature Science Foundation of China under Grant No. 61572328 and research funding of China-UK Visual Information Processing Lab.

### Conflict of interest statement

The authors declare that the research was conducted in the absence of any commercial or financial relationships that could be construed as a potential conflict of interest.
